# A lethal model of *Leptospira* infection in hamster nasal mucosa

**DOI:** 10.1371/journal.pntd.0010191

**Published:** 2022-02-22

**Authors:** Jiaqi Wang, Wenlong Zhang, Zhao Jin, Yue Ding, Shilei Zhang, Dianjun Wu, Yongguo Cao

**Affiliations:** 1 Department of Clinical Veterinary Medicine, College of Veterinary Medicine, Jilin University, Changchun, People’s Republic of China; 2 Key Laboratory for Zoonosis Research, Ministry of Education, College of Veterinary Medicine, Jilin University, Changchun, People’s Republic of China; Medical College of Wisconsin, UNITED STATES

## Abstract

Leptospirosis is a fatal zoonosis caused by contact between skin or a mucosal surface and contaminated soil or water. Hamsters were infected by intraperitoneal injection fto establish experimental leptospirosis, which is not a natural route of infection. There are no reports of nasal mucosal infection in hamsters. In this study, infection of the nasal mucosa was performed to establish a model of natural infection. Both methods of infection can cause lethal models with similar symptoms in the later stages of infection, such as weight loss, blood concentration, increased neutrophils (GRAN), and decreased lymphocytes (LYM) in the blood, severe organ damage and liver function obstruction. The burden of *Leptospira* in the organs and blood was lower in the mucosal inoculation groups at 1 day after infection. However, mucosal infection induced a higher *Leptospira* burden in urine than intraperitoneal infection in the late stages of infection. After nasal mucosal infection, antibody levels were higher and lasted longer. These results indicated that the route of nasal mucosal infection is a good choice for studying leptospirosis in hamsters.

## Author summary

The establishment of a leptospirosis experimental model is still key to elucidating the pathogenesis of leptospirosis. Hamsters were infected by intraperitoneal injection to establish experimental leptospirosis, although this is not a natural route of infection. The transmission characteristics of *Leptospira* and the disease progression in hamsters infected by a natural transmission route (e.g. through mucosal surfaces) had not been explored. In this study, we compared the dynamics of *Leptospira* infection in hamsters inoculated via the nasal mucosa or by intraperitoneal inoculation, and compared the burden of *Leptospira* and the level of antibodies produced with disease progression, such as body weight, serology, haematological changes and histopathological changes. Our data suggested that there are significant differences in the dynamics of infection between intraperitoneal and mucosal infection pathways. Although the result was the same in the later stage of infection, the course of mucosal infection was slower, which may better recapitulate the natural history of the disease, assist in studying kidney disease caused by *Leptospira*, and provide an animal model for the study of leptospirosis mucosal immunity.

## Introduction

Leptospirosis is a worldwide zoonotic disease. It is estimated that there are 1.03 million cases of this disease and 60,000 deaths each year [1,2]. In recent years, the number of outbreaks has increased with increases flooding and urbanization, as well as the the deterioration of living conditions of slums [3]. Leptospirosis occurs in humans through contact with infected urine from animals in daily activities, or by being in contact with contaminated soil or water [4–6]. Human leptospirosis ranges from a mild form to a severe infection called Weil’s disease, which is characterized by jaundice, renal failure and haemorrhage with a fatality rate of 5–15% [7]. In animals, *Leptospira* infection also causes reproductive failure and acute febrile disease with renal and liver failure [8,9]. The establishment of a leptospirosis experimental model remains key to elucidating the pathogenesis of leptospirosis. Hamsters or guinea pigs have been reported as animal models in most studies exploring the pathogenicity of *Leptospira*, because infection with virulent *Leptospira* can lead to fatal acute diseases in these species, similar to severe human leptospirosis [2,10].

In animal models of leptospirosis, including hamsters and guinea pigs, subjedts were typically infected by intraperitoneal (IP) injection which, because it is not a natural route of infection, ignores natural mucosal and skin defence mechanisms, with the result that this infection pathway may overestimate transmission time and pathogen load during transmission. Other routes of infection, such as contact between contaminated soil or water and epidermal, conjunctival, subcutaneous, intradermal, oral, intracardiac, and intracranial surfaces have also been reported [11,12], while detailed infection processes have been described only for intraperitoneal and subcutaneous intradermal routes of infection [12,13]. However, the assessment of leptospirosis progression in hamsters through the nasal mucosal infection route, which is an important natural transmissionpathway, remains elusive. The development of animal models for replication of natural transmission pathways is essential for understanding host pathogen interactions especially in the early stages of *Leptospira* infection.

The transmission characteristics of *Leptospira* and the disease progression of hamsters infected by natural transmission routes (e.g., through the mucosal surface) remain to be explored. Our purpose was to analyse similarities and differences between *Leptospira* transmission through the nasal mucosa and standard intraperitoneal transmission. In this study, we compared the dynamics of *Leptospira* infection in hamsters infected via the nasal mucosa and intraperitoneal routes, and compared the burden of *Leptospira* and the level of antibodies produced with disease progression, in addition to changed in body weight, serology, haematology changes and histopathology.

## Materials and methods

### Ethics statement

All animals were maintained on standard rodent chow with water supplied ad libitum and with a 12/12h light/dark cycle during the experimental period. All animal experiments followed the regulations for the Administration of Affairs Concerning Experimental Animals in China. The protocol was approved by the Institutional Animal Care and Use Committee of Jilin University (20170318).

### *Leptospira* and animals

*Leptospira interrogans Lai type Lai* (56601) and *Java type* (56602) are gifts from Professor Guo Xiaokui. *Australian* (56607) type and *Wolffel* (56635) type strains are cultivated in our laboratory all year round. *Leptospira* was grown in liquid Ellinghausen-McCullough-Johnson-Har- ris (EMJH) medium at 29°C. The virulence of the *Leptospira* was maintained by passage in hamsters. *Leptospira* was passaged *in vitro* less than three times in liquid EMJH for all infection studies. Before infection, the concentration of *Leptospira* was determined using a Petroff-Hausser counting chamber and a dark-field microscope. *Syrian golden hamsters* (Mesocricetus auratus) were provided by the Liaoning chang sheng biotechnology co. LTD.

### Hamster infection and sample collection

Intraperitoneal infection was performed as described previously [14]. Female hamsters obtained from the Animal Center of Jilin University, 5 to 6 weeks of age, were inoculated (1 mL) with 10^6^/10^7^/10^8^/10^9^ leptospires on day 0. *Leptospira* was counted by using a Petroff-Hausser chamber under a dark field microscope and confirmed by quantitative PCR (qPCR). For nasal mucosa (NM) infection, a maximum of 40μl of sterile saline containing ~10^6^/10^7^/10^8^/10^9^ spirochetes was deposited as small drops into one nostril of anesthetized hamsters, synchronized with inhalation. Body weights were monitored daily. After challenge with leptospires, all hamsters were observed no less than 3 times per day for a period of 21 days, during which serious sickness mouse appeared moribund was observed and then was humanely euthanized by CO_2_. Regarding sample collection, three hamsters in each group were humanely euthanized every day for the first five days, and then three hamsters in each group were humanely euthanized on 7/9/10/15 days. At every point in time, urine, blood and organs of hamster were collected. Blood was collected for routine blood test and chemical analyses (Jilin University Animal Hospital). Organs were collected for hematoxylin and eosin (H&E) staining and DNA extraction. Urine was collected for DNA extraction. Surviving hamsters were humanely euthanized after 21 days using CO_2_.

### Histopathology

Organs were collected and fixed with 4% paraformaldehyde for 24 hours at room temperature and then embedded in paraffin and sectioned at a thickness of 4 μm. Pathological changes of organ slices were examined by hematoxylin and eosin (H&E) staining, and the organ injury index (the injured area/total organ area x100%) was calculated for each slice. The severity of leptospire induced lesions was graded as previous description [14,15].

### Leptospiral load and qPCR assay

The leptospiral burden in organs, blood and urine of hamsters was determined by quantitative PCR (qPCR). The DNA extraction was followed as previously described [16]. The DNA concentration was measured by spectrometry. The primers used were specific for the lipL32 gene as described previously [17]. The qPCR assays were performed using previously published primers, optimized reaction mixtures and cycling parameters. A standard curve analyzed by serial dilutions (10^9^–10^2^) of DNA was established to calculate the number of *Leptospira* in the organ. Leptospiral load was expressed as the amount of genome equivalents per μg tissue DNA [14].

### Anti-leptospira ELISA

*Leptospira* was collected by centrifuging at 12,000 g for 5 min, after which *Leptospira* was resuspended in PBS and coated overnight at 4°C in 96-wells plates at a density of 10^7^. Then the plate was blocked with PBS-5% BSA at room temperature for 1 hour, washed, and a 15-fold dilution of hamster serum was added to the plate and kept at room temperature for 2 hours. After washing, HRP-conjugated goat anti-hamster IgG、IgG1、IgG2/3 and IgM were added for1h at room temperature. The plates were washed and peroxidase activity was revealed by TMB substrate (Sigma, USA), and stopped by H_2_SO_4_. Reading was performed at 450 nm. The titres of the sera were expressed as the dilution corresponding to twice the background level of the ELISA [18]. Alternatively, same dilutions of the different sera were also compared at 450 nm.

### Serum Microscopic Agglutination test (MAT)

All sera were tested by MAT. Serum water bath (56°C, 30min) was used to remove complement, then 15-fold dilution, and then continuous dilution of 8 times. The serum was mixed with an equal amount of 10^7^ 56601/56602/56607/56635. The mixture was incubated at 37°C for 2 hours and observe the agglutination under a dark field microscope. The antibody titers were recorded as the maximum dilution of serum with 50% agglutination or 50% reduction of *Leptospira* [19].

### Inhibition of Serum on growth of *Leptospira*

The serum were collected 15 day after infection, diluted 15 times and mixed with 10^7^ *Leptospira* 56601 in EMJH. Then the mixtures were incubated at 37°C for 2 hours. The growth of *Leptospira* was observed by microscopy daily.

### Statistical analysis

Survival differences between the study groups were compared by using the Kaplan-Meier log-rank test. Comparisons between groups were performed by using t-test. Differences were considered significant at P< 0.05.

## Results

### Nasal mucosal infection causes hamsters death

**Fig 1:**
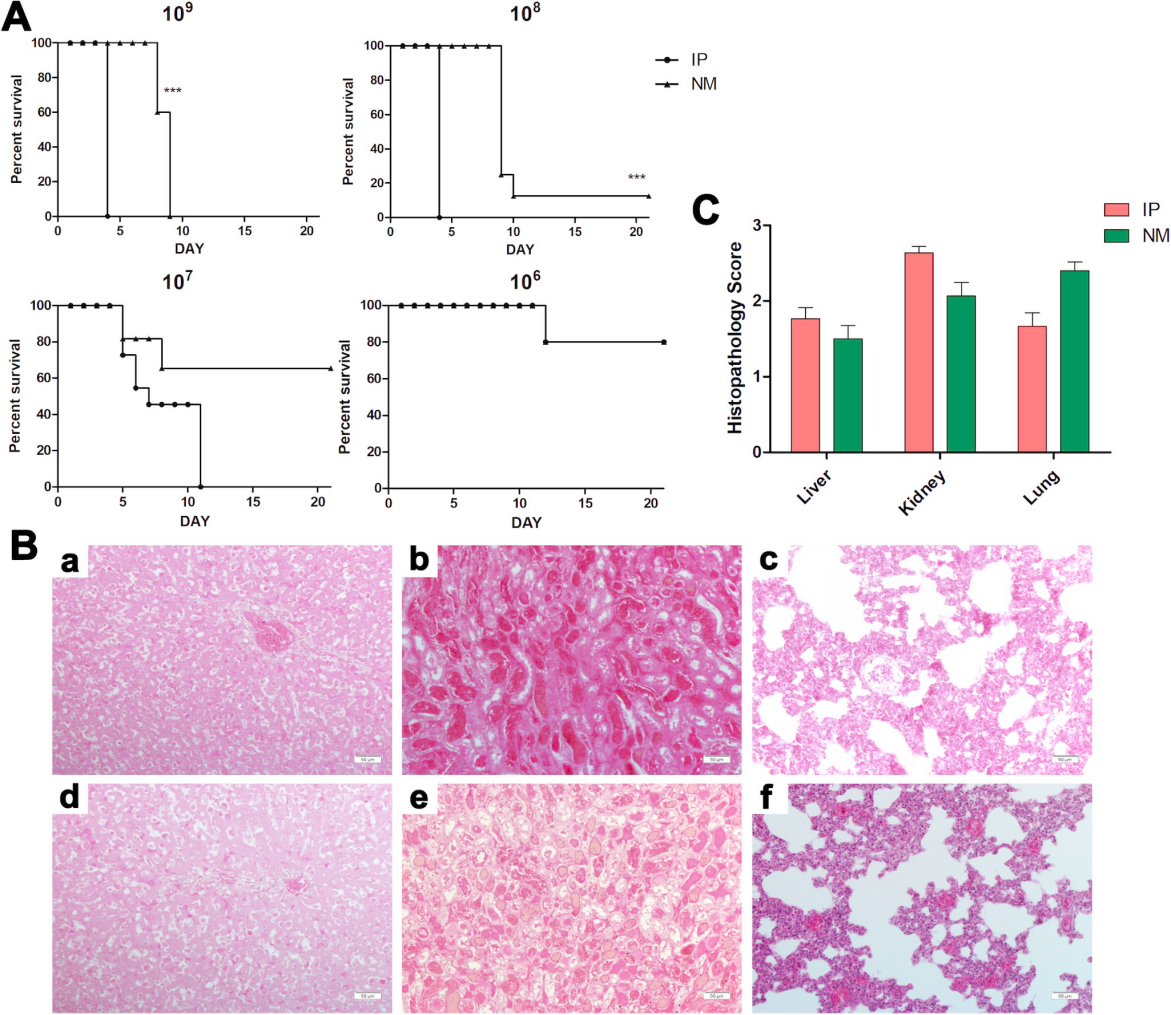
Pathogenic manifestations of different infection concentrations. (A) Survival curves of hamsters in the intraperitoneal(IP) group (n = 8) and the nasal mucosa (NM) group (n = 8). (B) Histopathology of the livers (a and d), kidneys ((b and e), and lungs (c and f) of hamsters in the IP group (a, b and c) and the NM group (d, e and f) infected with 10^8^ *Leptospira*. Magnification 200×. Samples were collected when the specimens appeared moribund, and representative images are shown. Statistical analysis of the results for the IP group (n = 3) and the NM group (n = 3) was performed by using the Wilcoxon rank sum test. (C) Histopathology scores of the livers, kidneys and lungs in hamsters.

Both methods of infection can establish lethal models. The median lethal dose (LD_50_) for intra-abdominal infection was 1.375×10^6^, and the LD_50_ for nasal mucosal infection was 1.29×10^7^ (Fig 1A). Representative photographs of hamster livers, kidneys and lungs were selected during the dying period (Fig 1B). Pathological changes in the livers of the three groups were tight junction defects and areas of necrosis and inflammatory infiltration (Fig 1Ba and 1Bd). Dramatic lesions with haemorrhage were found in the renal tissues (Fig 1Bb and 1Be). Haemorrhage was also observed in pulmonary tissues. Generalized interstitial pneumonia was noted in the lungs, alveolar congestion and infiltration of mononuclear cells (Fig 1Bc and 1Bf). The histopathological scores of the two groups were similar (Fig 1C).

### The late stage of nasal mucosal infection can simulate leptospirosis in sensitive animals, similar to intraperitoneal injection infection

**Fig 2:**
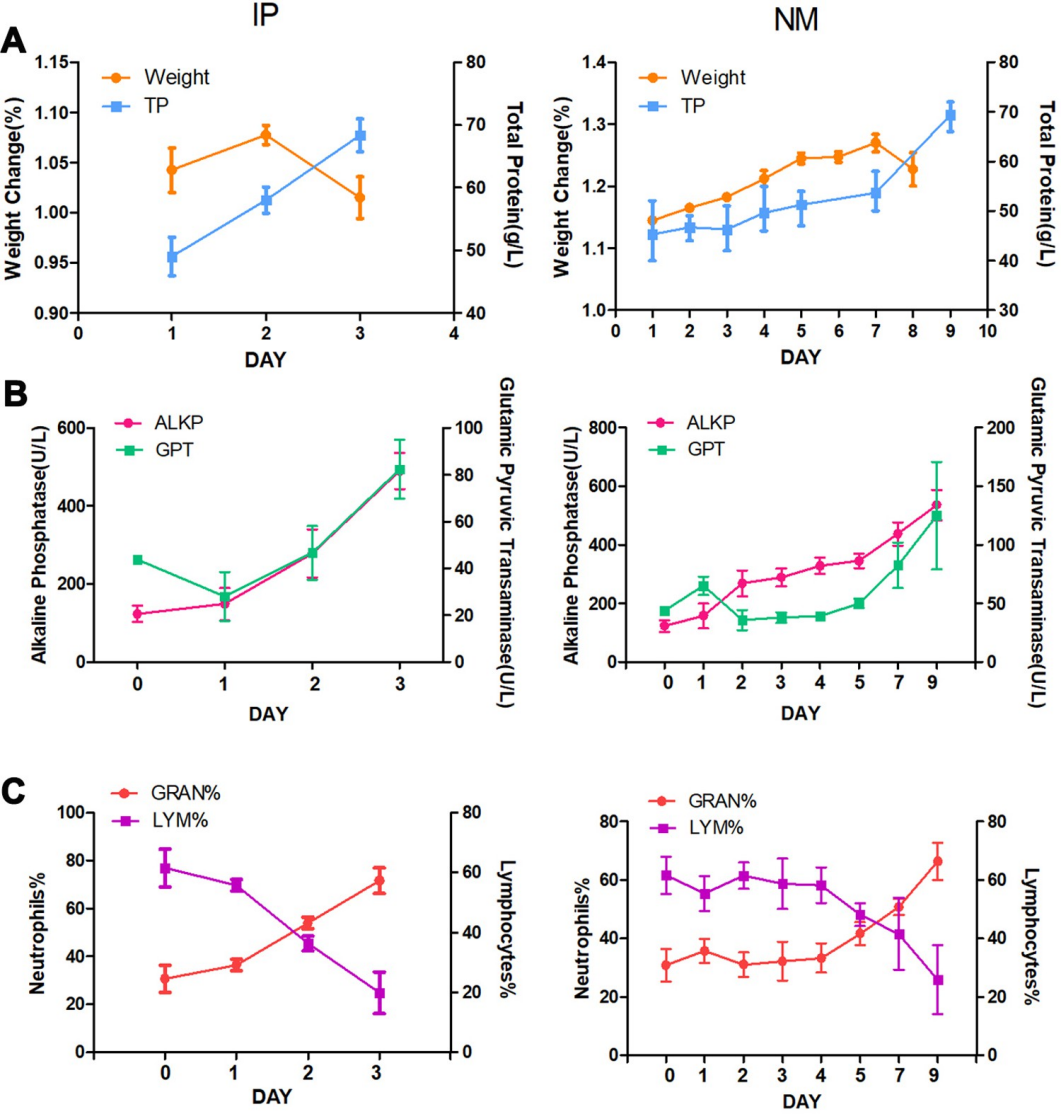
Hamster symptoms after 10^8^ *Leptospira* infections initiated via intraperitoneal and mucosal infection paths. (A) The weight of hamsters after infection. Animals were weighed at the time of IP and NM challenge (Day 0) and daily thereafter. The figure also shows the mean change in weight relative to the original weight and the amount of total protein in the serum. (B) Kinetics of inflammatory markers and liver enzymes. Alkaline Phosphatase (ALKP) and Glutamic Pyruvic Transaminase (GPT). (C) Neutrophils% and lymphocytes%.Blood samples were collected at the time of IP and NM challenge (Day 0) and daily thereafter.

The indicators of the two groups of hamsters infected with *Leptospira* 10^8^ were monitored. Infected hamsters continued to gain weight until the day before death. The weight of the hamsters in the IP group was reduced during the dying period by more than 5% compared with the previous day, and all of them died at 4 d p.i. At 9 d p.i., 75% of the hamsters with NM challenge had lost ≥5%, and these hamsters only survived to 9 d.p.i. The total protein content in the serum increased with the severity of the disease. The data show that the two infection routes had the same trend (Fig 2A).

After infection with *Leptospira*, both alkaline phosphatase (ALKP) and glutamic pyruvic transaminase (GPT) continued to increase. The intraperitoneal group reached its peak on the third day. The NM group peaked at 9 dpi (Fig 2B). During the late stage of infection, the percentage of neutrophils in the blood of hamsters of the both routes of infection increased significantly, from approximately 30% to approximately 60%, while the percentage of lymphocytes decreased significantly, from approximately 70% to 20% (Fig 2C).

**Fig 3:**
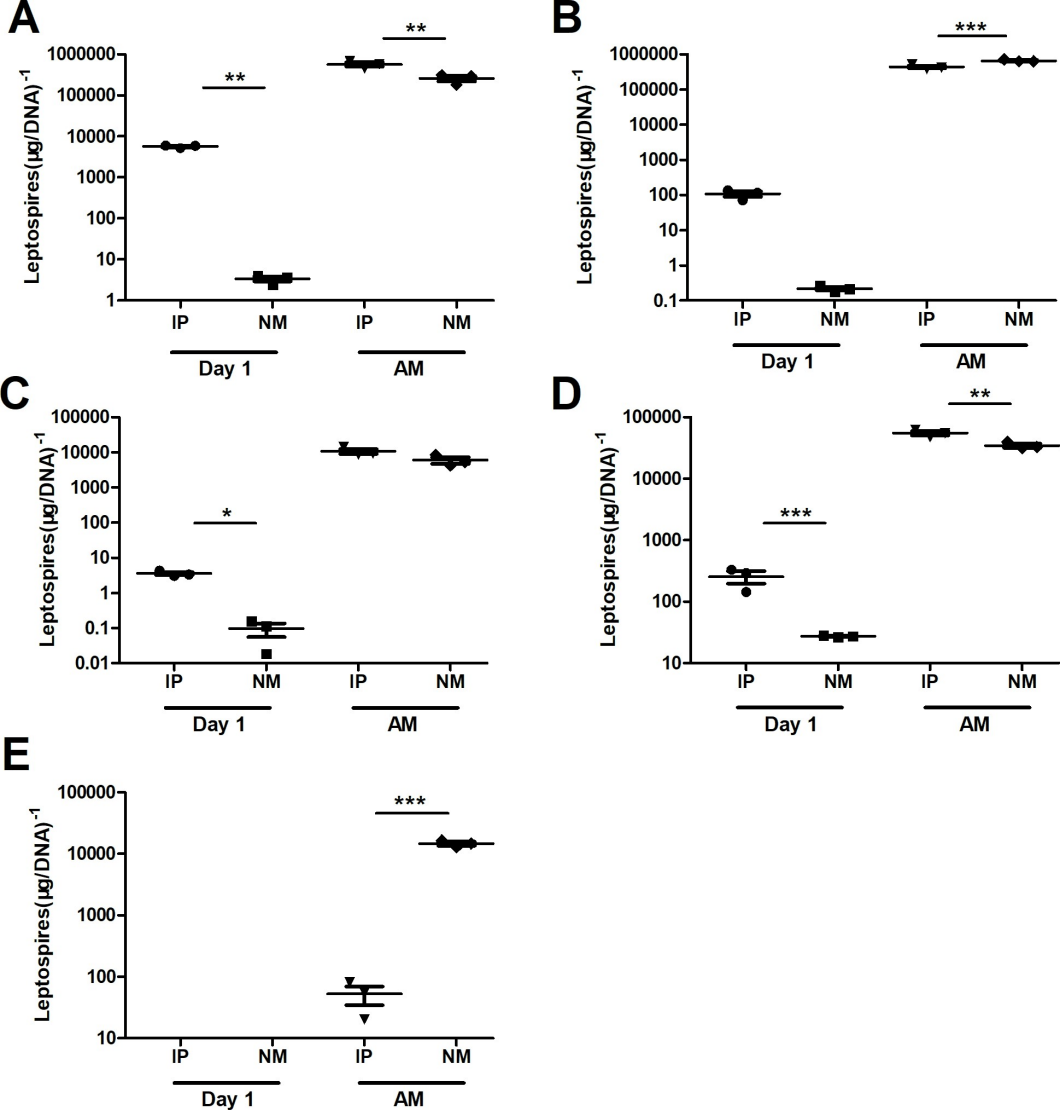
Leptospiral burdens in hamsters infected with 10^8^ *Leptospira*. Leptospiral burdens in the livers (A), kidneys (B), lungs (C), blood (D) and urine (E) of hamsters in the IP group (n = 3), the NM group (n = 3) at 1 d.p.i, and the day that they appeared moribund (AM) as determined by qPCR. Samples were collected on the 1st day after *Leptospira* infection and the day the hamsters appeared moribund. The results are presented as the number of genomic equivalents per microgram of tissue DNA, and the differences were compared by one-way ANOVA. *, P < 0.05.

*Leptospira* burden in the organs and blood of hamsters was detected at 1 d p.i. in different groups. The route of intraperitoneal infection allowed *Leptospira* to invade the hamster’s organs and blood faster (Fig 3A–3D). No *Leptospira* was detected in urine on the first day of infection (Fig 3E). During the dying period, large numbers of leptospires were found in organs and blood and the difference was not significant among the three groups (Fig 3A–3D). Interestingly, the *Leptospira* burden in the urine of the intraperitoneal group was much lower than thay in the mucosal group. The average amount of *Leptospira* in urine of the intraperitoneal group was 52, while the average amount of *Leptospira* in urine of the mucosal group was more than 10^5^ (Fig 3E).

Results for other infectious doses are in S1–S6 Figs.

### Nasal mucosal infections produce stronger and effective antibodies to fight the infection

**Fig 4:**
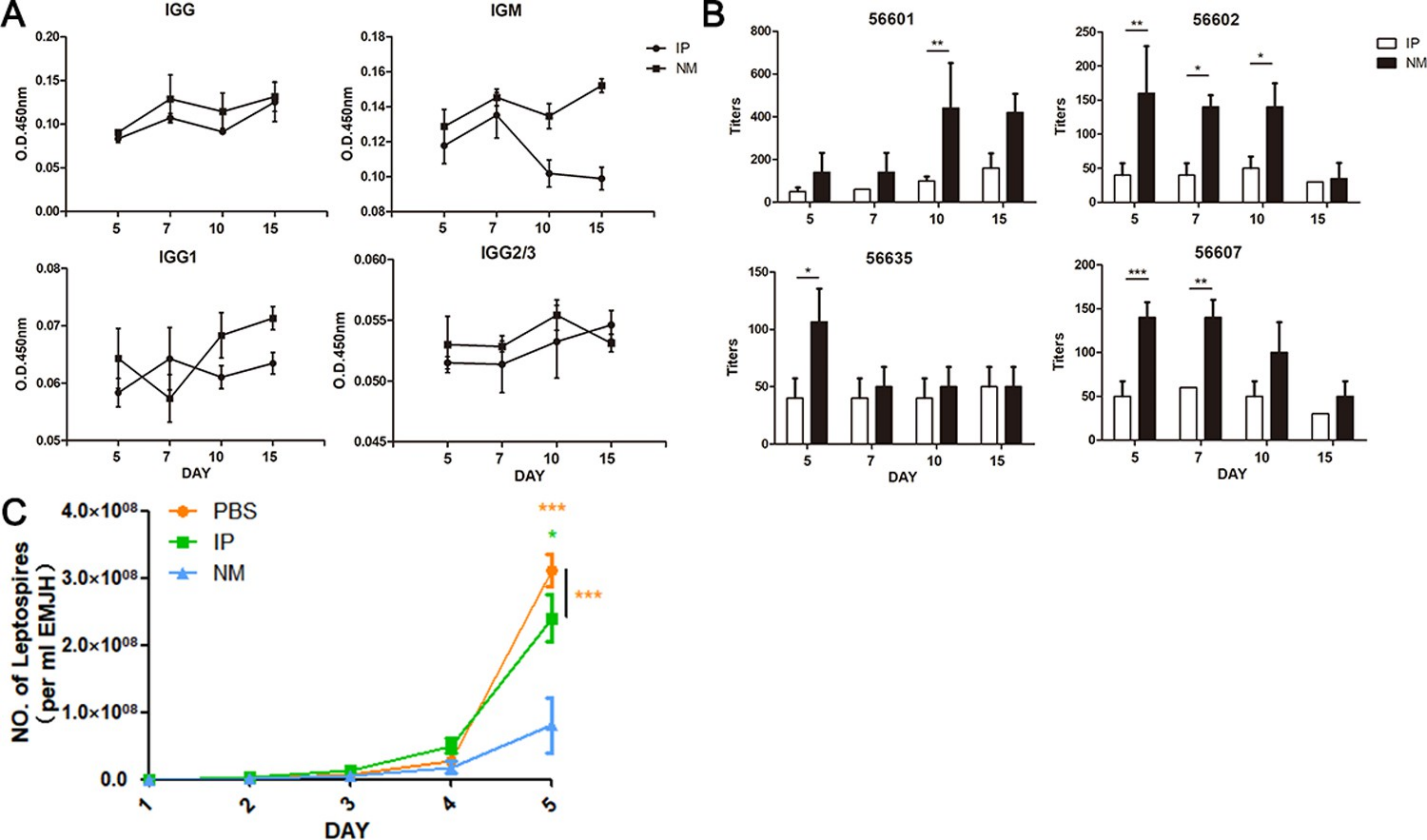
Antibody response in hamster infected with 10^6^ *Leptospira*. (A) Hamster immunoglobulin responses to heat-killed leptospires were measured by ELISA. Means are shown for serum samples (at a dilution of 1:15) obtained from hamsters challenged with nasal mucosal or intraperitoneal inoculation. (B) The serum was mixed with an equal amount of 10^7^ 56601/56602/56607/56635. The mixture was incubated at 37°for 2 hours and agglutination was observed under a dark field microscope. The antibody titres were recorded as the maximum dilution of serum with 50% agglutination or 50% reduction of *Leptospira*. (C) Place 50 μl with 10^7^ *Leptospira* and 50 μl serum from different infection routes at 29°C and incubate for 2 h. Thirty microlitres of the mixed solutionwas added to 3 ml EMJH for culture, and counted under a microscope every day.

The antibody levels of two groups of golden hamsters infected with *Leptospira* 10^6^ in the peritoneum and nasal mucosa were detected. The results showed that the serum IgG level after nasal mucosal infection was slightly higher than that of intraperitoneal infection, and IgG_1_ was significantly higher than that of intraperitoneal infection after 10 days of infection. IgM produced via nasal mucosal infection was higher than that produced by intraperitoneal infection, and IgM from intraperitoneal infection was significantly reduced after 10 days, while IgM produced by nasal mucosal infection continued to increase within 15 days (Fig 4A).

The MAT results with 56601/56602/56607/56635 showed that the antibody titre produced by nasal mucosal infection was higher, and the binding ability to the four kinds of *Leptospira* was stronger (Fig 4B).

The mixture of serum and 56601 wwa incubated for 2 hours in EMJH. Daily microscopic examination recorded the growth of *Leptospira*. After incubation with serum produced by the nasal mucosal infection route, *Leptospira* grew, and reproduction was significantly suppressed (Fig 4C).

## Discussion

Hamsters are currently one of the most widely used animal models of acute leptospirosis because of their reproducibility and sensitivity to multiple pathogenic *Leptospira* strains [20–22]. A feasible lethal model that is closer to the natural infection mode is more helpful to research on leptospirosis. There have been a few report on this aspect of the disease. Reports on mucosal infection pathways have used mice or rats as experimental animals [23,24]. The Albert I Ko team used hamsters to compare changes in the amount of *Leptospira* after infection of the conjunctiva and the abdominal cavity [25], but did not study the route of infection of the nasal mucosa and did not describe the mortality rate. However, nasal mucosal infection has been is the main method of inducing the disease and cannot be ignored. Studies have shown that oral mucosal infection of *Leptospira* does not cause hamster death. [26] Therefore, in this study, we think that inoculation via the nasal mucosa will be an effective and comprehensive method for modelling the disrasr. Our ultimate goal was to assess whether hamsters infected via the nasal mucosa could reproduce disease markers, histopathology, and other diseases previously caused by intraperitoneal infection.

Infection with different concentrations of *Leptospira* led to the death of hamsters, and the damage to the organs during the dying period was similar. However, at the same dose, mucosal infection had a slower course and a lower fatality rate than abdominal infection. The LD50 for intra-abdominal infection was 1.375*10^6^, and the LD50 for nasal mucosal infection is 1.29*10^7^. (Fig 1) This difference might be caused by a decrease in the number of Leptospires that cross the natural mucosal barrier or the rapid activation of mucosal immunity to clear a large number of leptospires present in the inoculum [27–29]. The epithelial layer consists of columnar ciliated and secretory cells that line the airway surface and help it serve as an environmental barrier for inhalation. These cells cover smaller basal cells, which have the characteristics of progenitor cells. They not only show the ability of self-renewal and clonal expansion in the homeostasis and epithelial repair process after injury, but also cause basal, ciliary and secretory lineages [29]. Secreted goblet cells combine with the submucosal gland to produce mucus, which contains hydrated gelatine and many host defence and cytoprotective molecules such as antimicrobial molecules, proteases and antioxidants [30].

However, this step may be absent in the route of intra-abdominal infection, which may explain why *Leptospira* strains deficient in genes that were shown to have important functions in vitro remain infectious during intraperitoneal inoculation of a living host [31–33]. The results from this study, which show differences in infection kinetics, suggest that it may be crucial to study the infectivity contribution of those genes using more physiologically relevant models, such as the mucosal route of infection.

Analysing the sample data of 10×LD_50_ infection, we found that when the weight is reduced by more than 5% from the previous day, daeth will result cause death (Fig 2). This finding may be used to predict or avoid death. Weight loss and total protein increased simultaneously, suggesting that these changes may be due to dehydration and blood concentration caused by leptospirosis, which may be due to reduced fluid intake and impaired sodium reabsorption in pr oximal renal tubules [34]. In addition, the identified histological changes were initially related to the glomeruli and interstitium, while the renal tubules were only affected in the late stage, which was the reason why the total protein increased rapidly in the late stage [35]. Elevated TP may have indicated inflammation in this study as TP is documented to increase due to hyperfibrinogenemia [36].

In the late stage of leptospirosis, the results of the three infection routes were similar. There were many leptospires in the liver and kidney, and pathological damage was severe (Fig 1). Jaundice was found when the abdominal cavity was opened, and serological examination also showed severe liver damage (Fig 2). Hamsters died quickly due to infection of the abdominal cavity. *Leptospira* was not detected in the urine, while the number of *Leptospira* in the urine of mucosal infections reached 10^4^ (Fig 3). This is consistent with the previous study by Nair et al. in mice [23]. Although *Leptospira* can also be detected in urine at the later stage of abdominal cavity infection, hamsters died prematurely from abdominal infection, and hamsters survived for a longer period with mucosal infection. *Leptospira* colonize the renal tybules of hamsters in the late stage of mucosal infection. Combined with pathological sections, kidney damage was serious. This is consistent with the rapid development of a hamster model similar to human leptospirosis, which directly leads to severe liver and kidney dysfunction [37,38]. A large number of leptospires could be detected in the blood, and haematological analysis found that the percentage of neutrophils increased and the percentage of lymphocytes decreased, which was a manifestation of severe sepsis [39,40].

Detection of serum antibodies in golden hamsters infected with 10^6^ *Leptospira* showed that nasal mucosal infections produced higher levels of antibodies than individuals with abdominal infections, especially after ten days, and the levels of IgM and IgG_1_ were significantly higher than those with intraperitoneal infections. Through MAT detection of the antibody titres that bind to the four serotypes of *Leptospira*, it was found that the antibodies produced by nasal mucosal infections have stronger binding ability to different serotypes of *Leptospira*, so the antibodies produced by nasal mucosal infection may have stronger capability (Fig 4).

The experimental results on hamsters are similar to those on rats and mice [11,23,24]. Oral mucosal infection does not cause infection, while infections through the conjunctiva and nasal mucosa can kill sensitive animals. In tolerance animal models, *Leptospira* can enter the blood and colonize the kidneys. This may be related to secretions in the oral cavity that differ from those in the nasal cavity and conjunctiva.

In summary, our data suggested that there are significant differences in the dynamics of infection between intraperitoneal and mucosal disease pathways. More leptospires may be required to overcome mucosal defence and transit through tissue in sufficient numbers before systemic spread can occur. Although the result was the same in the later stage of infection, the course of mucosal infection was slower, which that may better recapitulate the natural history of the disease, thereby better assisting evaluations of kidney disease caused by *Leptospira*, and providing an excellent animal model for the study of leptospirosis mucosal immunity. There there were differences in the immune pathways activated by the two paths of infection, which may yield different efficacy outcones in testing of vaccines and preventive drugs in these different hamster models of infection.

## Supporting information

S1 Fig Hamster symptoms after 10^9^ *Leptospira* infections initiated via intraperitoneal and mucosal infection paths. (A) The weight of hamsters after infection. Animals were weighed at the time of IP and NM challenge (Day 0) and daily thereafter. The figure also shows the mean change in weight relative to the original weight and the amount of total protein in the serum. (B) Kinetics of inflammatory markers and liver enzymes. Alkaline Phosphatase (ALKP) and Glutamic Pyruvic Transaminase (GPT). (C) Neutrophils% and lymphocytes%. Blood samples were collected at the time of IP and NM challenge (Day 0) and daily thereafter.
(TIF)

S2 Fig Leptospiral burdens in hamsters infected with 10^9^ *Leptospira*. Leptospiral burdens in the livers (A), kidneys (B), lungs (C), blood (D) and urine (E) of hamsters in the IP group (n = 3), the NM group (n = 3) at 1 d.p.i., and the day that theyappeared moribund (AM) as determined by qPCR. Samples were collected on the 1st day after infected *Leptospira* infection and the day the hamsters appeared moribund. The results are presented as the number of genomic equivalents per microgram of tissue DNA, and the differences were compared by one-way ANOVA. *, P < 0.05.
(TIF)

S3 Fig Hamster symptoms after 10^7^ *Leptospira* infections initiated via intraperitoneal and mucosal infection paths. (A) The weight of hamsters after infection. Animals were weighed at the time of IP and NM challenge (Day 0) and daily thereafter. The figure also shows the mean change in weight relative to the original weight and the amount of total protein in the serum. (B) Kinetics of inflammatory markers and liver enzymes. Alkaline Phosphatase (ALKP) and Glutamic Pyruvic Transaminase (GPT). (C) Neutrophils% and lymphocytes%. Blood samples were collected at the time of IP and NM challenge (Day 0) and daily thereafter.
(TIF)

S4 Fig Leptospiral burdens in hamsters infected with 10^7^ *Leptospira*. Leptospiral burdens in the livers (A), kidneys (B), lungs (C), blood (D) and urine (E) of hamsters in the IP group (n = 3), the NM group (n = 3) at 1 d.p.i, and the that they day appeared moribund (AM) as determined by qPCR. Samples were collected on the 1st day after infected *Leptospira* infection and the day the hamsters appeared moribund. The results are presented as the number of genomic equivalents per microgram of tissue DNA, and the differences were compared by one-way ANOVA. *, P < 0.05.
(TIF)

S5 Fig Hamster symptoms after 10^6^ *Leptospira* infections initiated via intraperitoneal and mucosal infection paths. (A) The weight of hamsters after infection. Animals were weighed at the time of IP and NM challenge (Day 0) and daily thereafter. The figure also shows the mean change in weight relative to the original weight and the amount of total protein in the serum. (B) Kinetics of inflammatory markers and liver enzymes. Alkaline Phosphatase (ALKP) and Glutamic Pyruvic Transaminase (GPT). (C) Neutrophils% and lymphocytes%. Blood samples were collected at the time of IP and NM challenge (Day 0) and daily thereafter.
(TIF)

S6 Fig Leptospiral burdens in hamsters infected with 10^6^ *Leptospira*. Leptospiral burdens in the livers (A), kidneys (B), lungs (C), blood (D) and urine (E) of hamsters in the IP group (n = 3), the NM group (n = 3) at 1 d.p.i, and the that they day appeared moribund (AM) as determined by qPCR. Samples were collected on the 1st day after infected *Leptospira* infection and the day thehamsters appeared moribund. The results are presented as the number of genomic equivalents per microgram of tissue DNA, and the differences were compared by one-way ANOVA. *, P < 0.05.
(TIF)
