# Supplementary figures and images for: A lethal model of Leptospira infection in hamster nasal mucosa
Source: PLoS Negl Trop Dis. 2022 Feb 22;16(2):e0010191. doi: 10.1371/journal.pntd.0010191 (PMC8863242; doi:10.1371/journal.pntd.0010191)

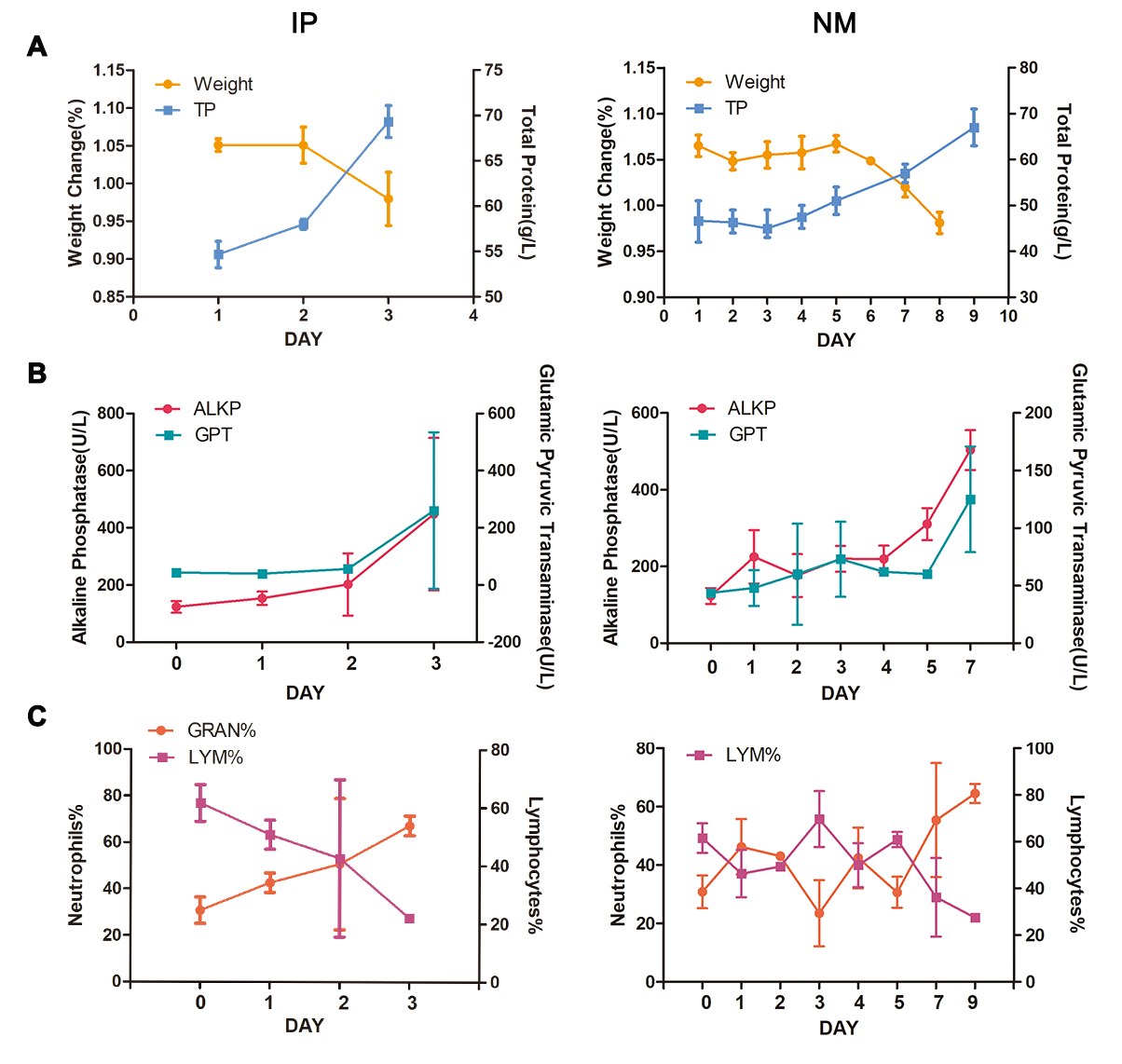

Supplement: S1 Fig — (A) The weight of hamsters after infection. Animals were weighed at the time of IP and NM challenge (Day 0) and daily thereafter. The figure also shows the mean change in weight relative to the original weight and the amount of total protein in the serum. (B) Kinetics of inflammatory markers and liver enzymes. Alkaline Phosphatase (ALKP) and Glutamic Pyruvic Transaminase (GPT). (C) Neutrophils% and lymphocytes%. Blood samples were collected at the time of IP and NM challenge (Day 0) and daily thereafter. (TIF) [file pntd.0010191.s001.tif]

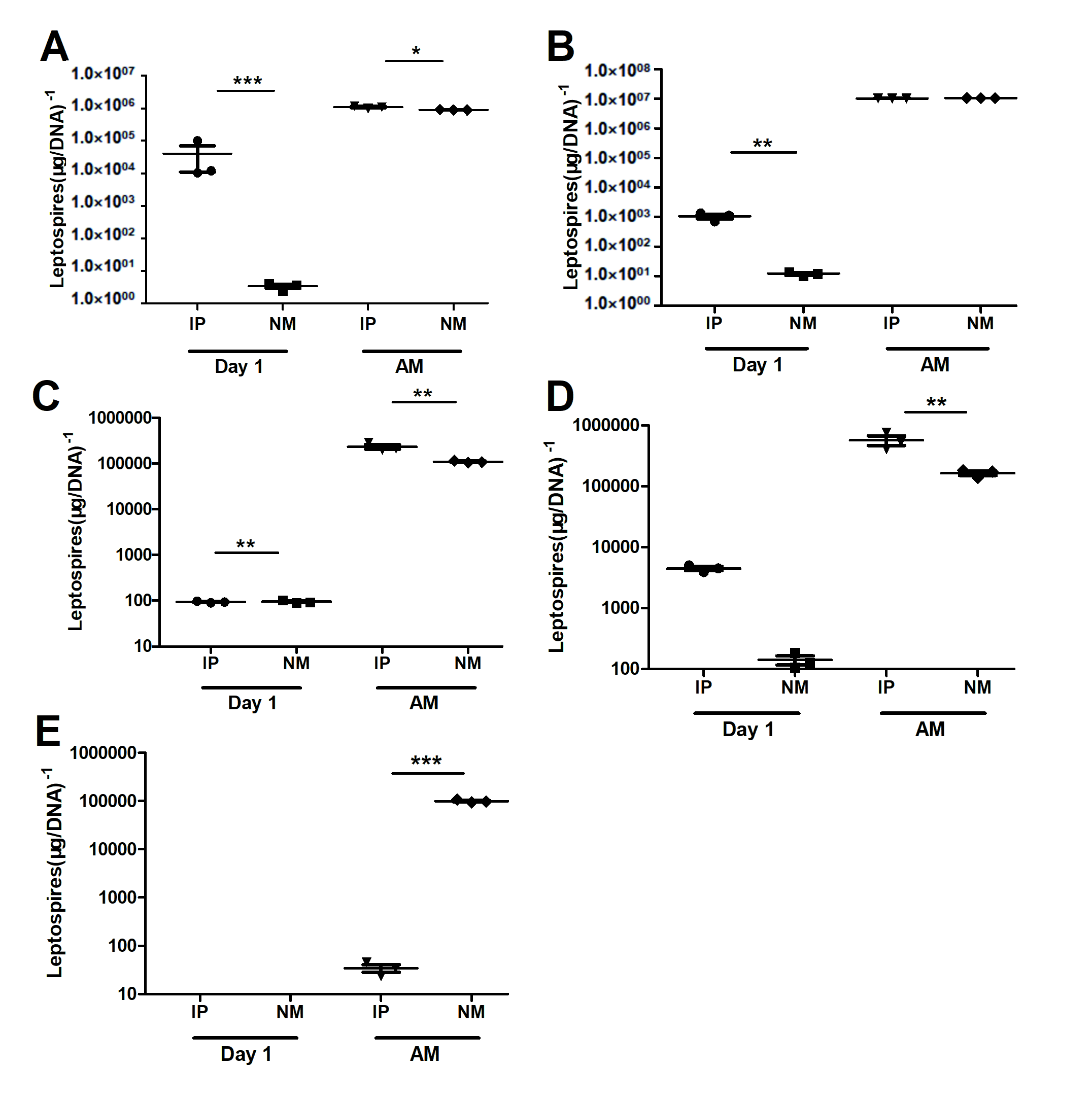

Supplement: S2 Fig — Leptospiral burdens in the livers (A), kidneys (B), lungs (C), blood (D) and urine (E) of hamsters in the IP group (n = 3), the NM group (n = 3) at 1 d.p.i., and the day that theyappeared moribund (AM) as determined by qPCR. Samples were collected on the 1st day after infected Leptospira infection and the day the hamsters appeared moribund. The results are presented as the number of genomic equivalents per microgram of tissue DNA, and the differences were compared by one-way ANOVA. *, P < 0.05. (TIF) [file pntd.0010191.s002.tif]

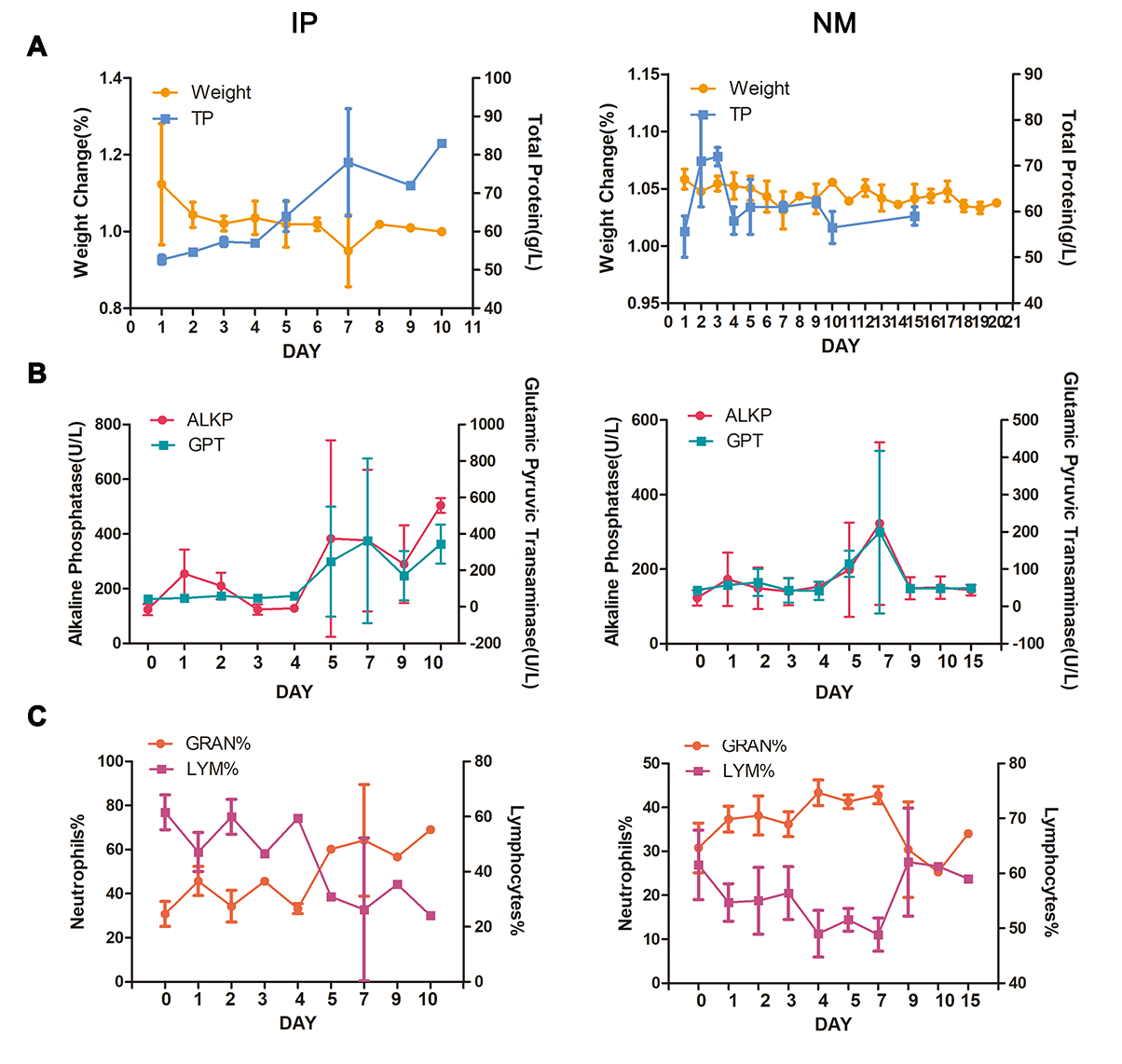

Supplement: S3 Fig — (A) The weight of hamsters after infection. Animals were weighed at the time of IP and NM challenge (Day 0) and daily thereafter. The figure also shows the mean change in weight relative to the original weight and the amount of total protein in the serum. (B) Kinetics of inflammatory markers and liver enzymes. Alkaline Phosphatase (ALKP) and Glutamic Pyruvic Transaminase (GPT). (C) Neutrophils% and lymphocytes%. Blood samples were collected at the time of IP and NM challenge (Day 0) and daily thereafter. (TIF) [file pntd.0010191.s003.tif]

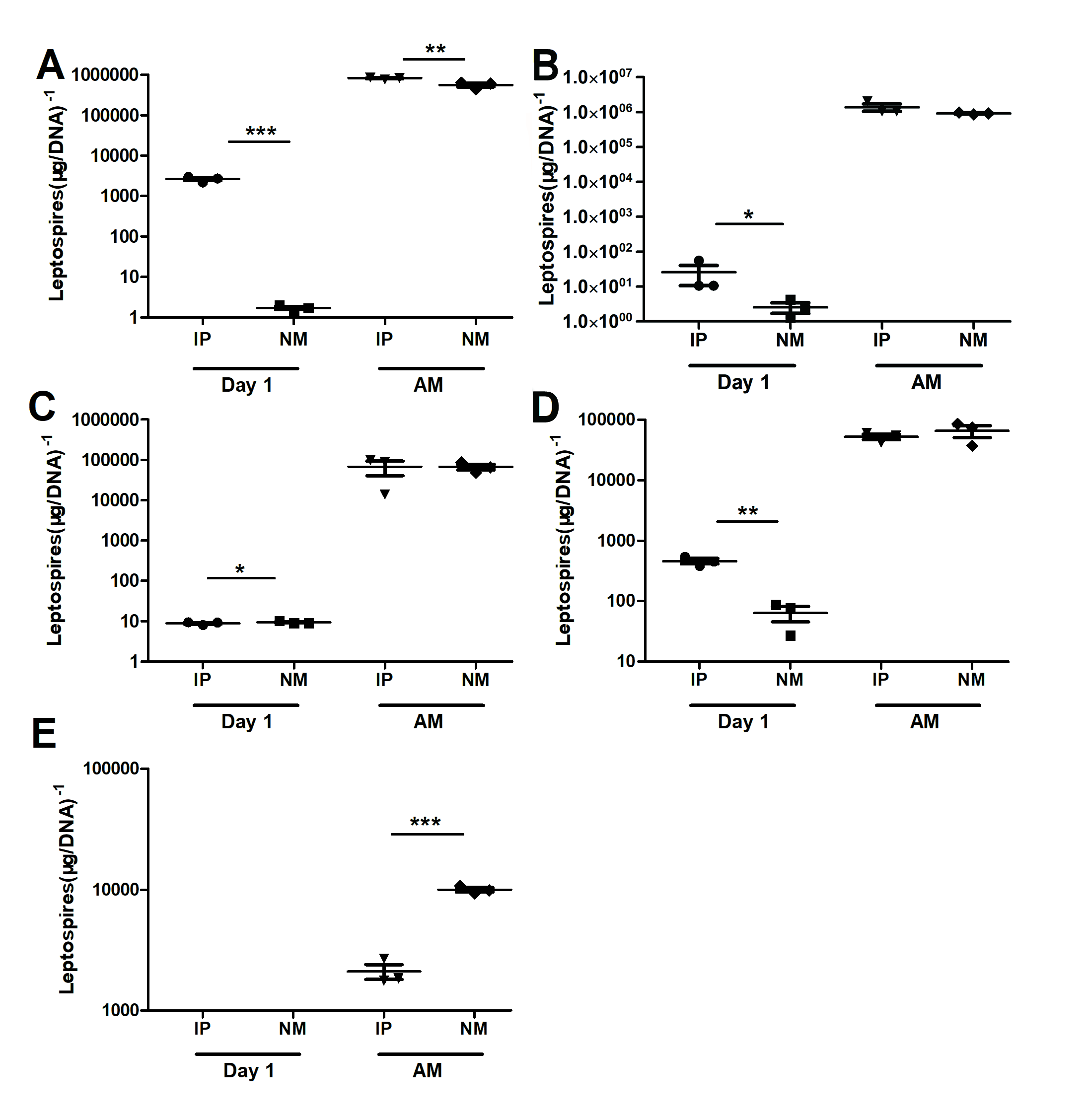

Supplement: S4 Fig — Leptospiral burdens in the livers (A), kidneys (B), lungs (C), blood (D) and urine (E) of hamsters in the IP group (n = 3), the NM group (n = 3) at 1 d.p.i, and the that they day appeared moribund (AM) as determined by qPCR. Samples were collected on the 1st day after infected Leptospira infection and the day the hamsters appeared moribund. The results are presented as the number of genomic equivalents per microgram of tissue DNA, and the differences were compared by one-way ANOVA. *, P < 0.05. (TIF) [file pntd.0010191.s004.tif]

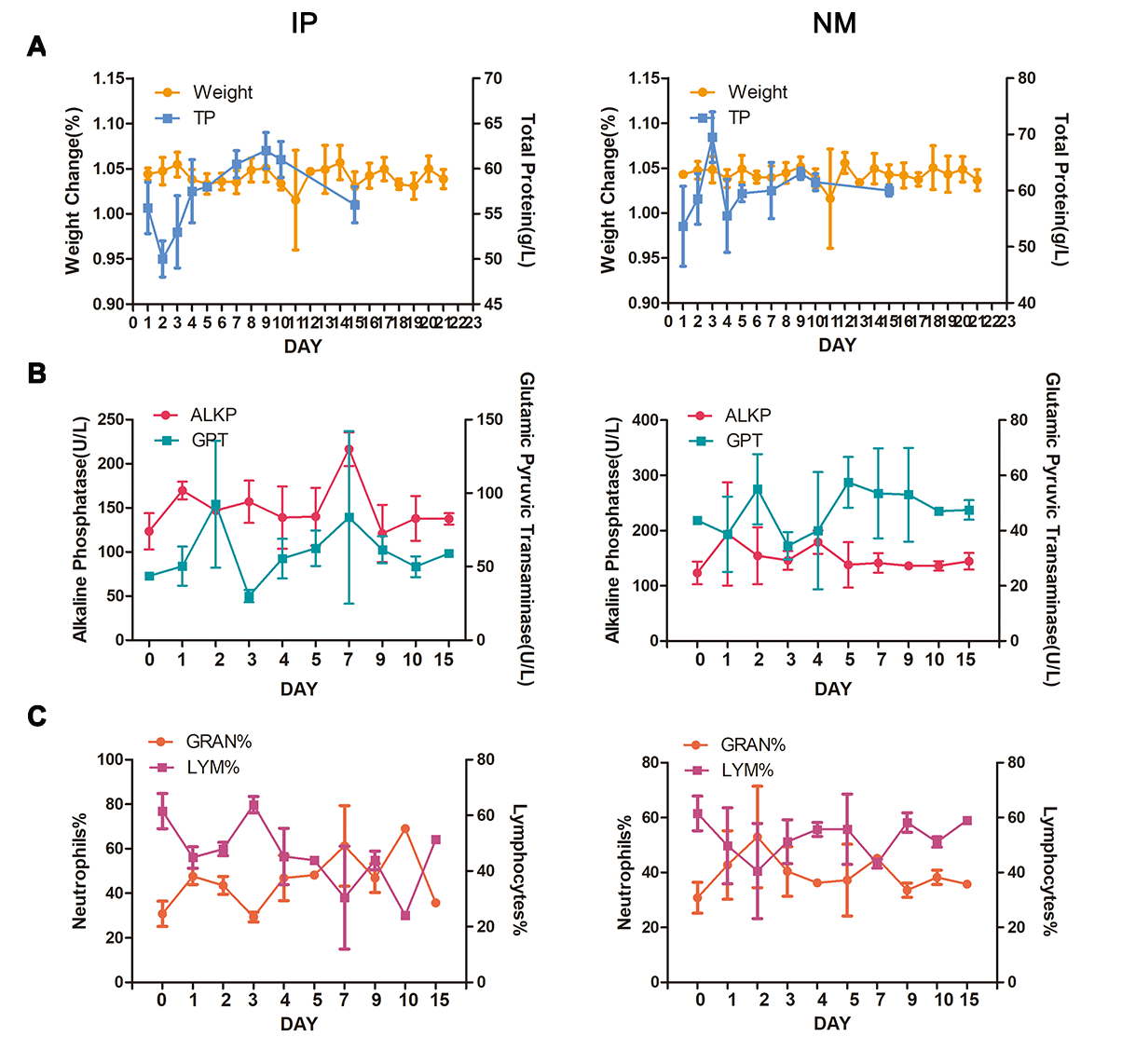

Supplement: S5 Fig — (A) The weight of hamsters after infection. Animals were weighed at the time of IP and NM challenge (Day 0) and daily thereafter. The figure also shows the mean change in weight relative to the original weight and the amount of total protein in the serum. (B) Kinetics of inflammatory markers and liver enzymes. Alkaline Phosphatase (ALKP) and Glutamic Pyruvic Transaminase (GPT). (C) Neutrophils% and lymphocytes%. Blood samples were collected at the time of IP and NM challenge (Day 0) and daily thereafter. (TIF) [file pntd.0010191.s005.tif]

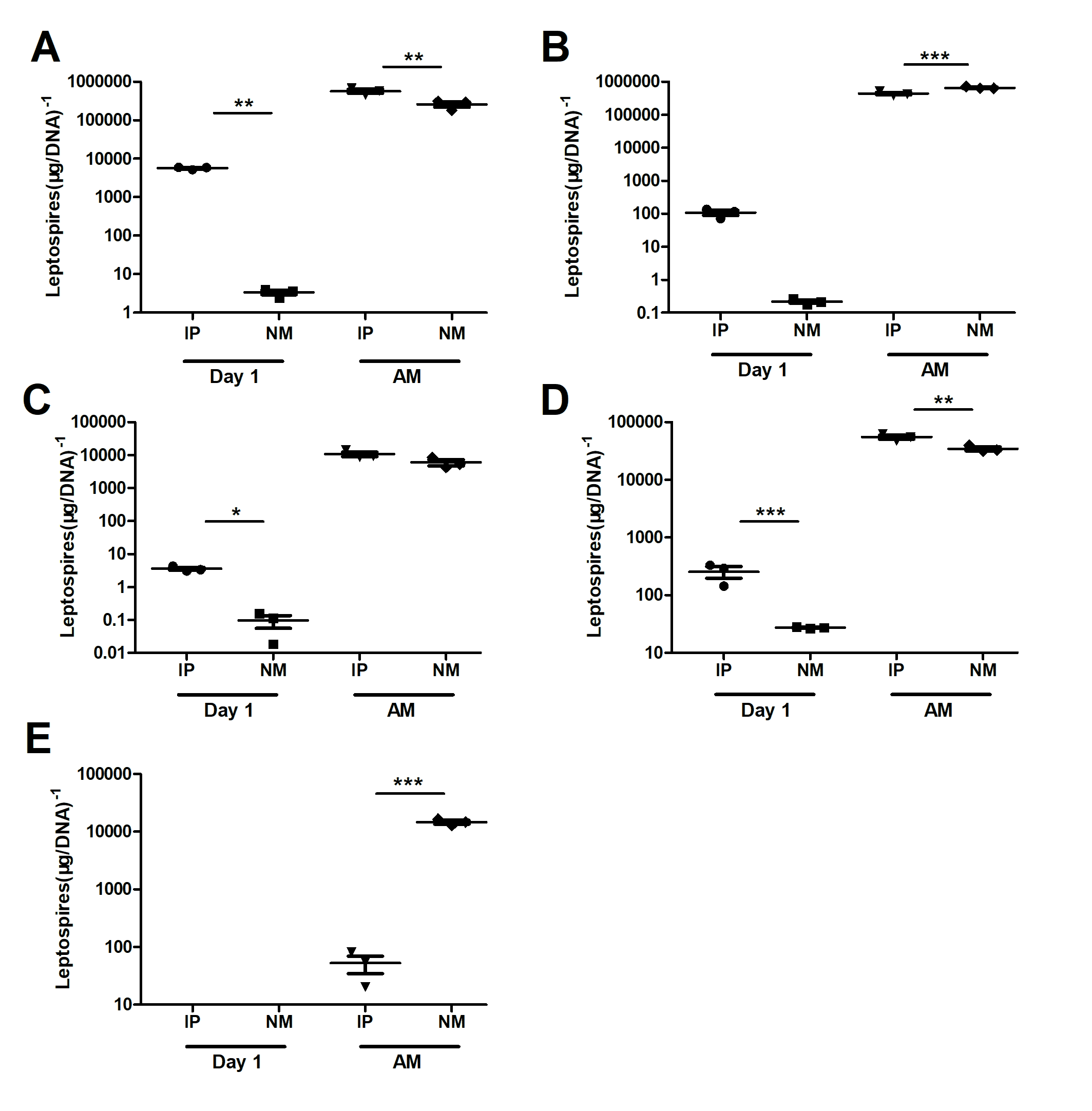

Supplement: S6 Fig — Leptospiral burdens in the livers (A), kidneys (B), lungs (C), blood (D) and urine (E) of hamsters in the IP group (n = 3), the NM group (n = 3) at 1 d.p.i, and the that they day appeared moribund (AM) as determined by qPCR. Samples were collected on the 1st day after infected Leptospira infection and the day thehamsters appeared moribund. The results are presented as the number of genomic equivalents per microgram of tissue DNA, and the differences were compared by one-way ANOVA. *, P < 0.05. (TIF) [file pntd.0010191.s006.tif]
